# From gene to treatment: supporting rare disease translational research through model systems

**DOI:** 10.1242/dmm.039271

**Published:** 2019-02-22

**Authors:** Julija Hmeljak, Monica J. Justice

**Affiliations:** 1 Disease Models & Mechanisms, The Company of Biologists, Bidder Building, Station Road, Histon, Cambridge CB24 9LF, UK; 2 Program in Genetics and Genome Biology, The Hospital for Sick Children, and Department of Molecular Genetics, The University of Toronto, Toronto, Ontario M5G 0A4 Canada

**Keywords:** Rare disease, Disease model, Face validity, Genome editing, Clinical translation

## Abstract

Individual rare diseases may affect only a few people, making them difficult to recognize, diagnose or treat by studying humans alone. Instead, model organisms help to validate genetic associations, understand functional pathways and develop therapeutic interventions for rare diseases. In this Editorial, we point to the key parameters in face, construct, predictive and target validity for accurate disease modelling, with special emphasis on rare disease models. Raising the experimental standards for disease models will enhance successful clinical translation and benefit rare disease research.

## Abstract (precis)

**Summary:** This Editorial discusses the importance of model systems with accurate face, construct, target and predictive validity for rare disease research.

## Introduction

Disease Models and Mechanisms (DMM) is a journal dedicated to improving human health through publishing research in model systems. To this end, DMM publishes articles that develop and validate models for translational research, as well as research that would aid in the diagnosis or treatment of human disease. This unique position makes DMM a highly relevant venue for preclinical research in rare diseases. Rare diseases are particularly difficult to recognize, diagnose and treat. Additionally, the definition of a rare disease differs among countries, with the European Commission defining conditions that affect fewer than 1 in 2000 people as rare (https://ec.europa.eu/health/non_communicable_diseases/rare_diseases_en), whilst the US National Institutes of Health considers those conditions that affect fewer than 200,000 people to be rare (https://rarediseases.info.nih.gov/diseases). Rare diseases are usually genetic in origin, and cover a spectrum of causes from mutations in metabolic enzymes to those affecting immune or muscle function, or those that cause cancer. Rare diseases therefore span a wide range of phenotypes, affecting nearly any organ system or biological function. Most rare diseases affect fewer than one person in 100,000, yet, when a new condition is recognized and defined, new diagnoses may swiftly change the number of patients affected by a particular disease condition. Currently, 6000-8000 rare diseases have been identified so, as a whole, they affect a large percentage of the population. Because rare disorders as a group are common yet, singly, may affect only a few individuals, they are very difficult to understand and eventually treat by studying humans alone. Moreover, developing treatments for any disease is very expensive, and profit margins are small when a new treatment is applied to a rare disorder, making them unlikely to be a priority for drug development. For rare diseases, model organisms are required to validate genetic associations, to understand gene function and to develop therapeutic interventions. This Editorial reflects on recent research on rare diseases published in DMM. In honour of Rare Disease Day on February 28, we have assembled a special collection that covers recent publications in DMM that inform rare diseases, showing how model organisms reveal new gene functions, are used in preclinical studies, and enable the screening of novel and repurposed treatments.

**Fig. 1.**
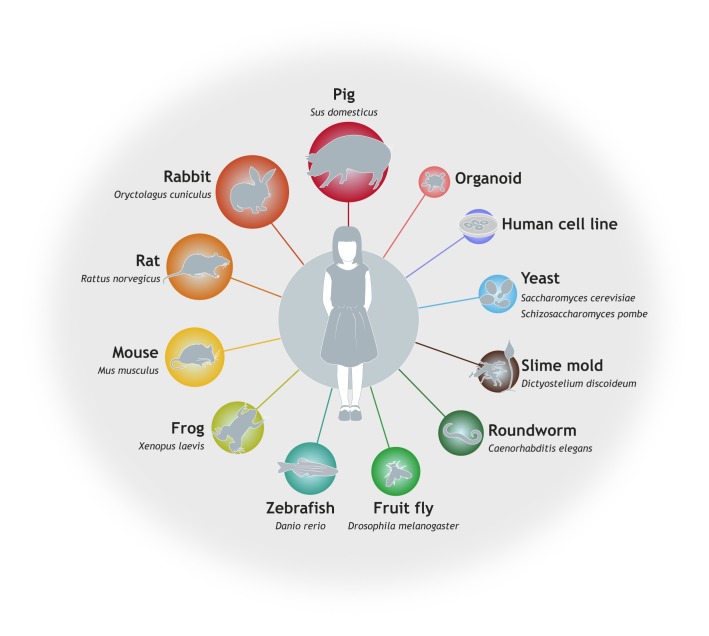
**Model systems used in rare disease research.** Researchers can use a variety of model systems, spanning from simple *in vitro* cell lines to large animals, to infer rare disease mechanisms, identify gene networks and therapeutic targets, and to test drugs. Each model system has both advantages and pitfalls, and model choice depends on a careful assessment of the model's face, construct, predictive and target validity.

A strength of DMM is its breadth of model system coverage, which can be particularly valuable to the rare disease field. Our special collection includes papers describing studies in yeast, slime mold, worms, flies, fish, mice, rats, rabbits, pigs and human cell lines (Fig. 1). When developing a model for mechanistic or translational studies, both genetic conservation and validity in predicting the behaviour of the human disease need to be considered. Models that have been rigorously validated to model human disease are more likely to be considered at DMM.

## Choosing a model system

Determining which model to use – cells, organoids, yeast, worms, flies, fish, mice or larger animals – may depend on the cost, speed and tools available for assessing phenotype parameters in the organism, while considering its validity as a model for human disease. Each model system has unique advantages and limitations. The ability to generate many units quickly and carry out manipulations are key factors in modelling disease.

Cell lines are amenable to screening using antibiotic or growth supplements, making them ideal for high-throughput technologies, but lack the physiological outcomes provided by whole organisms (Joung et al., 2017). Organoids, or multi-dimensional culture systems, solve many of the issues that arise in a monolayer cell culture, but have issues with variability (Hurtado Del Pozo et al., 2018; Phipson et al., 2019). Yeast has simple diploid genetics, and can be grown on media that use nutritional supplements for selection and analysis at a single-cell level (Smith and Snyder, 2006). The nematode worm's full developmental lineage from a single cell to an adult animal has been traced, making it extremely well suited for mechanistic genetic studies (Jorgensen and Mango, 2002). The fruit fly has a short generation time along with the most extensive genetic toolkit (St Johnston, 2002), allowing the application of many approaches to model human diseases (Ugur et al., 2016).

Although yeast, worms and flies are advantageous because of their well-curated genomes supported by exhaustive online databases and powerful tools for genetic analyses, they are evolutionarily divergent from the human, such that gene content, and sometimes function, is not conserved. Many disease-associated phenotypes occur in zebrafish mutants (Patton and Zon, 2001), yet much of the zebrafish genome is duplicated, making functional redundancy an issue for the interpretation of experimental results. Therefore, in lieu of genetic screens, high-throughput chemical screening for drug discovery is particularly amenable to the fish habitat and experimentation (Strahle and Grabher, 2010; Tseng et al., 2018). Because of its gene conservation, similar mammalian characteristics and the ability to engineer nearly any human mutation, the mouse has emerged as a key tool in preclinical testing for drug and drug target discovery (Zuberi and Lutz, 2016). Often, a multi-system approach is ideal to model and understand a human disease, and can be extremely valuable for rare diseases where human tissues are difficult to obtain. Whatever its strengths or restrictions, a good model system will have face, construct, predictive and target validity for the disease of interest.

## Face and construct validity

Although models for disease may have ‘face’ similarities in anatomical, physiological and/or behavioural phenotypes to the human condition, they should also have construct validity. For the best models, the gene target should have similar genetics and mechanism to the human disease gene and, if possible, should be an orthologue of the human gene. Ideally, the gene will have amino acid and sequence conservation, expression in similar cell types, be controlled by a similar regulatory mechanism and function in a similar pathway or have similar downstream targets (see Target validity). Experimental autoimmune encephalomyelitis (EAE) in mice is commonly used as an experimental model for the human inflammatory demyelinating disease multiple sclerosis (MS). MS is a complex disease with unknown aetiology that may involve multiple genes. EAE shares some features with MS, including the presence of T cells that cross the blood-brain barrier to target the myelin sheath, inducing neuroinflammation, demyelination and, sometimes, death of axons. However, EAE can have many phenotypes depending on the mouse strain, the myelin antigens used to induce the autoimmune response and the mode of induction (Hasselmann et al., 2017). Although its outward neurological and immunological phenotypes mimic some that occur in MS, this model has ‘face’ but not ‘construct’ validity. Therefore, treatments developed from this model are likely to ameliorate the downstream symptoms rather than treat the cause of the human disease.

Recapitulating the precise mutations found in a human disease has the strongest predictive and preclinical application. Precise mutations can now be generated in nearly any model organism by adapting the bacterial-innate RNA-guided immune-response CRISPR/Cas system to genome editing. In the past, the genetic tools available for yeast, worms, flies and mice allowed them to rise to the experimental forefront. Now, zebrafish have particularly benefited from the advent of Cas endonuclease-driven genome editing. Three DMM papers in the special collection describe the generation of precise genetic models of human diseases, exploit error-prone repair after endonuclease activity and insert mutagenic cassettes into specific cells using the CRISPR/Cas system (Tessadori et al., 2018; Boel et al., 2018; Callahan et al., 2018). These techniques have greatly expanded the repertoire for genetic experimentation in the fish and will undoubtedly empower rare disease modelling. However, RNA-guided genome editing is not limited to small organisms; instead, it may be used to generate larger animal models that may provide better preclinical representation for drug and therapy delivery (Perleberg et al., 2018). In this special collection, several articles show the use of rabbits and miniature pigs to develop disease models for skeletal dysplasias and neurological disorders that provide more phenotypic similarities to the human disease than those developed in the mouse. A new model of Duchenne muscular dystrophy developed by CRISPR/Cas genome editing in the rabbit has more similar clinical features to the human than a genetically valid mouse model (Sui et al., 2018; Wells, 2018).

## Predictive validity

An accurate genetic model must also have ‘predictive’ validity, i.e. a clinical intervention should have a similar outcome as it does in the human, since many factors such as genetics, physiology and environment may influence the results. Therefore, an important step in testing a new model is to assess its response to a known treatment. In an infamous case of failed preclinical studies in amyotrophic lateral sclerosis (ALS), a mouse model of TDP43-ALS (Wegorzewska et al., 2009), was used in preclinical drug testing, yet the drugs that were successful in the mice ultimately failed in clinical trials (Perrin, 2014). Subsequent analyses showed that the mouse model had different primary phenotypes to the human condition, not mimicking the progressive muscle weakness characteristic of ALS. Instead, smooth muscle failure led to bowel obstruction in TDP43-ALS mice (Hatzipetros et al., 2014). This example highlights how a model's relevance to the human disease being studied, supported by strong data, is crucial.

DMM's subject collection includes, among others, four manuscripts that highlight the predictive validity of small, evolutionarily distant organisms for modelling human disease and performing candidate drug screening. The first, on a rare dopamine-serotonin vesicular transport disease caused by mutations in the *SLC18A2* gene, describes a *Caenorhabditis elegans* model with construct validity (Young et al., 2018). The authors investigated the therapeutic effects of serotonin and pramipexole, two drugs used in the clinic, demonstrating that serotonin could successfully restore a pharyngeal pumping phenotype and showing predictive validity of this system for developing and assessing treatments for this rare disease. In a similar study, a NemaFlex system for testing muscle strength in the worm was used to test different genetic models of muscular dystrophy and their response to drugs. Two compounds, prednisone and melatonin, which are used in the clinic, were then used to determine the degree of improvement and mitochondrial response in the most severe model, validating this worm model for further screening of novel candidate drugs (Hewitt et al., 2018). Similarly, Delerue et al. (2019) used a mutant fission yeast model in a drug-repurposing screen to identify compounds that could rescue mitochondria, the failure of which is a hallmark of optic atrophy and other inherited mitochondrial diseases.

The examples above highlight how the predictive validity of a model system should be tested by ensuring that the model responds in the same way to drugs that have been used in the human. This step in model validation is essential prior to testing novel candidate drugs. Indeed, for submission to DMM, the predictive value of a newly developed model should be tested.

## Target validity

Finally, a model should have ‘target’ validity, i.e. the molecular targets of the root gene should be the same in the model and in the human, as should the regulation and downstream molecular effects of the disease-causing gene variant. This is always a concern in model systems that may be less complex than the human, even when a model is ‘humanized’ to carry the human gene sequence, sometimes with the precise disease-associated mutation (Slijkerman et al., 2018). For a rare disorder like cystic fibrosis, the conservation of the entire functional pathway and affected organ systems between human and mice is very high, making treatments in the mouse an accurate representation of the likely response in the human (Knowles and Durie, 2002). However, in other genetic disorders, the gene's function and its downstream targets may be vastly different due to evolutionary divergence. Often, a gene's downstream targets may be validated by proteomics or transcriptomics. For example, the special collection includes an article describing a mouse model for a rare allergic disease called eosinophilic esophagitis (EoE) whose phenotype strongly models the human disease (Eden et al., 2017). Mouse gene expression datasets were compared with those of humans with EoE, and confirmed by protein studies, demonstrating target validity, and revealing a new role for non-canonical NF-κB signalling in the disorder. Even distantly related organisms may have target validity: a mutation in the *Drosophila* orthologue of the human mitochondrial citrate transporter (*CIC*; called *sea* in flies), reveals a metabolic link between citrate transport and oncometabolite accumulation, uncovering a mechanistic basis for symptoms that develop in CIC deficiency (Li et al., 2018).

Clearly, models with high target validity are especially valuable for mechanistic studies. Cultured human cells or organoids may provide the highest degree of target validity but often lack the ability to manipulate their genetics and environment, and cannot provide information on physiological interactions among organs. One solution to this issue is the development of model organisms that carry human cells, or organisms that carry precise human mutations. Such an approach had its beginning in cancer research, where leukaemia cells were transplanted into mice that lacked an appropriate immune system to reject the human cells, now called ‘patient-derived xenografts’ (PDX) (Geisse and Kirschbaum, 1950). However, PDX models often lack the heterogeneity seen in the primary tumour, and may not accurately represent the drug response (Holen et al., 2017). Here, Floc'h et al. have developed a computational tool for evaluating the predictive power of PDX models, allowing for a refinement in the numbers of animals used as it assesses treatment efficacy (Floc'h et al., 2018). Zebrafish researchers are also developing xenotransplant models: here, Allende and colleagues implanted mouse hematopoietic cells into fish embryos, providing an avenue for using the higher-throughput fish model as a screening tool for infectious disease (Parada-Kusz et al., 2018). Such studies pave the way for using fish as a carrier for human cells in chemical screening (Britto et al., 2018). Providing multiple layers of evidence through work on several model systems is the preferred approach to glean a more complete picture of the disease mechanism. This is especially important in the context of rare disease, where the phenotypic manifestations and natural history of disease may vary significantly within a small patient population.

## Expanding model organism research for rare diseases

The essential role of valid animal models in rare disease research is widely recognized in the community, and has resulted in new programmes to improve and extend rare disease animal modelling. The Rare Disease Models and Mechanisms (RDMM) network funded by the Canadian Institutes of Health Research aims to marry human disease researchers with model organism researchers to develop new models for diseases that will also validate the clinical discovery disease associations in the human. The generation of a model system may thus inform functional data, insights into pathogenesis and therapeutic targets. By fostering a collaboration between researchers, the RDMM aims to create a research network for rare diseases.

Many downstream functional assays in model systems reveal disease mechanisms and suggest pathways for treatment. The Model Organism Screening Center (MOSC) was established within the National Institutes of Health (NIH)-funded Undiagnosed Disease Network (UDN) to use fly, worm and zebrafish genetics to understand the pathogenesis of rare and undiagnosed diseases (Wangler et al., 2017). The MOSC may first investigate whether a unique variant identified in the genomes of rare disease patients may contribute to disease pathogenesis by altering the orthologous gene in the model organism. They may also use genetic and genomic technologies to define the functional pathways for orphan genes; one approach involves ‘humanizing’ the fly by expressing both the human reference and the variant cDNA in the model organism prior to additional genetic studies. This collection contains a manuscript that uses a CRISPR/Cas9 editing approach to replace genes in *C. elegans* with their human orthologues and the variant version (McDiarmid et al., 2018). Functional variants can also be recapitulated in fish using CRISPR/Cas9 genome editing (Tessadori et al., 2018). A rapid screen in a small organism may quickly decipher the functional consequences of the hard to interpret ‘variants of unknown significance’, which are often associated with human rare diseases.

All genetic diseases are characterized by variability in the onset and expression of clinical features, often due to second-site gene modifiers. Modifier genes are epistatic to the root-disease-causing gene, and their products often lie in the same functional pathway. For rare diseases that do not have power in patient populations, identifying gene modifiers in a model organism could accelerate the annotation of key pathways to identify entry points for therapeutics. Modifier screens have been adapted to many organisms, including cells, yeast, worms, flies and mice. Current screens cover a broad range of basic functional gene networks, as well as human genetic disease (Karim et al., 1996; Jaiswal et al., 2012; Feany, 2000; Carpinelli et al., 2004; Buchovecky et al., 2013). In a modifier screen, a second-site variant may change the phenotypic presentation of the gene under study. Modifier screens involve the analysis of many individuals to confirm the ability to change the phenotype and subsequently identify the causative gene. Such screens are more commonly performed in organisms with short generation times and ease of maintenance. A genetic screen in a mutant budding yeast strain identified myosin- and calcium-dependent calmodulin signalling as potential drug targets in the rare neurodegenerative disease chorea-acanthocytosis (Soczewka et al., 2019). Zacchi et al. performed a genetic screen in yeast for factors that would modify the levels of the torsinAE protein, which is associated with early-onset torsion dystonia, a rare muscle disorder (Zacchi et al., 2017). Thus, an organism without muscles was used to identify pathway members involved in a muscle disease.

Following on the idea that root genes may be modified, small organisms such as yeast, worms, flies and fish are particularly well adapted to chemical screening for drug discovery (Strange, 2016). Such screens have become a powerful method for treating complex diseases such as rare cancers. The concept of synthetic lethality, wherein concomitant loss of function of two genes is fatal, but loss of only one of them is not, is a commonly assessed outcome in fly and worm screens for genes that act in a common pathway and to identify new treatment targets (Sonoshita and Cagan, 2017). In one example, flies that co-express a mutant form of Ras along with an inhibiting RNA against PTEN die as larvae due to defects in the trachea that model lung cancer. A screen discovered two compounds that synergistically suppressed lethality and tumour formation, whose efficacy was confirmed in human cells (Levine and Cagan, 2016). Together, these studies demonstrate that systems that do not fully model the human disease phenotype remain useful in assessing the underlying mechanisms of disease, providing critical functional annotation of the affected gene or identifying other genes in the disease pathway that may be more amenable to producing more accurate animal models, or to therapeutic interventions.

Additional programmes will reveal information for linking the unusual phenotypes seen in rare diseases with genetic causes. The Knockout Mouse Project (KOMP) is annotating gene function in the mouse by systematically knocking out every gene in the mouse genome, examining expression (Tuck et al., 2015) and carrying out a broad-based phenotype assessment on cohorts of mice from each gene knockout (Justice, 2008; Adissu et al., 2014; Brown et al., 2018). Many of the alleles generated by the consortium are conditional-ready, allowing for the spatial or temporal removal of the gene from a subset of cells. In hereditary sensory and autonomic neuropathy (HSAN) type III, the IKBKAP protein plays an important role in sensory and autonomic function, resulting in the death of patients by the age of 40 (Slaugenhaupt et al., 2001). A KOMP allele of *IKBKAP* was used to remove the gene specifically from the central nervous system (CNS), revealing that IKBKAP plays an essential role in the survival of both cortical and motor neurons (Chaverra et al., 2017). The KOMP project has demonstrated, through the analysis of thousands of mouse mutations, how both lethal and viable mutations in mammalian genes can reveal new causal associations with rare diseases (Dickinson et al., 2016; Meehan et al., 2017). A website hosted by the International Mouse Phenotyping Consortium (IMPC; www.mousephenotype.org), will allow for additional phenotypes and/or genes to be linked to a rare disease (Brown and Moore, 2012). Ultimately, these data are an invaluable resource in our quest in understanding disease processes and developing innovative treatments.

Although modifier screens and similar high-throughput approaches could potentially be performed in cell-based models, model organisms are essential to gauge the efficacy and potential side effects of a treatment in the whole organism; moreover, comprehensive phenotyping in whole animals can dissect out the basis for the clinical signs as it parses the effects of a treatment. Although the advantages of small model organisms in mechanistic and drug discovery studies are clear, translating the delivery of therapies in a small organism such as the mouse to a large organism such as the human has led to disappointing results in clinical trials (Modi and Sahin, 2018; Perrin, 2014). For all model systems, it is important to set high standards that will increase experimental reproducibility and the likelihood that findings will successfully translate to the clinic (Justice and Dhillon, 2016). In this regard, larger animals may provide a better platform for drug delivery and dosage studies. The ability to introduce precise mutations using CRISPR-Cas genome editing allows for organisms such as rats, rabbits or pigs to be used as genetically valid preclinical models as well. Model organism research may also aid clinical trial design by informing, for example, the importance of the timing of treatment, and by helping to identify prognostic and predictive biomarkers. Studies of muscle cells *in vitro* show that pharmacological targeting of pathways that act at a specific stage of myogenic differentiation may provide therapeutic benefit for a rare form of muscular dystrophy (Collins et al., 2017). Combining this knowledge with stage-specific dosage studies in mice and/or large animals would validate the findings and provide a solid foundation for translation into the clinical setting.

For rare diseases in particular, model systems will increasingly be used as platforms for understanding the mechanistic basis for the natural history of the disease, providing essential knowledge for therapy development and clinical trial design. DMM has served as a hub for community communication of translational work in model systems, and will remain a forum for publishing rare disease research. The rapid and open-access publication model means that research published in DMM is freely available to the rare disease community of researchers, policymakers and health professionals, as well as patients, their families and their advocates.
